# Unusual Presentation of a Parotid Gland Malignancy: A Case Report

**DOI:** 10.7759/cureus.68253

**Published:** 2024-08-30

**Authors:** Gurmehr Singh, Soya Alfred Xavier, Karthikeyan Ramalingam, Murugesan Krishnan

**Affiliations:** 1 Oral and Maxillofacial Surgery, Saveetha Dental College and Hospitals, Saveetha Institute of Medical and Technical Sciences, Saveetha University, Chennai, IND; 2 Oral Pathology and Microbiology, Saveetha Dental College and Hospitals, Saveetha Institute of Medical and Technical Sciences, Saveetha University, Chennai, IND

**Keywords:** rare tumors, salivary gland tumor, salivary gland surgery, mucoepidermoid carcinoma (mec), parotid malignancy

## Abstract

The most commonly occurring malignant salivary gland tumor is mucoepidermoid carcinoma (MEC). It consists of intermediate cells, squamous cells, and mucous-secreting cells. It is usually not capsulated and is identified by mucocarmine staining. Mucoepidermoid carcinoma exists in the thyroid gland and lungs as well. This report presents a case of a very rare sclerosing variant of MEC of the parotid gland in a 48-year-old patient. The patient presented with a small swelling below the left earlobe. Ultrasound-guided fine-needle aspiration cytology (FNAC) was carried out. A diagnosis of pleomorphic adenoma was given. The patient underwent a partial parotidectomy under general anesthesia. The final diagnosis was made through histopathological examination after the surgical removal of the tumor. The patient is now under close follow-up to look out for a recurrence. This case highlights the importance of recognizing and managing rare variants of MEC to optimize patient outcomes.

## Introduction

Mucoepidermoid carcinoma (MEC) was first described by Berger and Masson in 1924 [1]. Since then, a large number of cases of MEC have been reported and described. Its prevalence is seen to be higher in women than men in the ratio of 3:2 [2]. The age group most commonly affected by this is in the third to fifth decade. Out of all the malignancies seen in the major and minor salivary glands, around 35% of them are diagnosed as MEC. It is the most common salivary gland malignancy among children.

Mucoepidermoid carcinomas usually present as painless, slow-growing growth that is generally firm and hard on palpation. It is usually seen in the parotid gland but can also be found in the submandibular gland and minor salivary glands. The symptoms vary from patient to patient. Some experience facial pain, muscle spasms, facial paralysis, or simply tenderness over the long-standing mass. Some patients have also reported fluid draining from the ears and difficulty with deglutition, whereas some have experienced no symptoms at all. Their only chief complaint is an asymptomatic mass below their earlobe. Intraorally, MEC usually presents as bluish-red swelling and is often fluctuant in nature. In such cases, it can be easily confused with either a mucocele or even a vascular malformation. Depending on the cellular structure, tumor formation is divided into several types or constructs, such as low-grade MEC, intermediate-grade MEC, high-grade MEC, clear-cell MEC, sclerosing MEC, and oncocytic MEC [3]. 

This report presents a case of a sclerosing variant of MEC in a 48-year-old male. Despite the common diagnosis of MEC among the malignancies, the sclerosing variant is in fact a very rare occurrence. It was first documented and described in 1987 by Chan and Saw [4]. Since then, only 32 cases have been documented. It is a type of salivary gland tumor that arises from the mucous and epidermoid cells in the salivary glands. It is characterized by the presence of abundant hyalinized or sclerotic stroma surrounding the tumor cells. This fibrous or sclerotic stroma can sometimes be so prominent that it can obscure the tumor cells, making diagnosis challenging. This variant of MEC has a high incidence of recurrence and a relatively poor prognosis. Hence, the patients should be kept on a strict and regular follow-up schedule.

Boahene et al. [5] have done a retrospective clinical and histopathological review of 128 cases of MEC involving the parotid gland. They have reported that with adequate parotidectomy and appropriate neck dissection, the prognosis was very good with very few recurrences, irrespective of the tumor grading and tumor staging. Sclerosing MEC is a rare variant, and only six cases have been reported in the oral cavity [6]. In this case report, we present a malignant tumor involving the parotid gland.

## Case presentation

**Figure 1:**
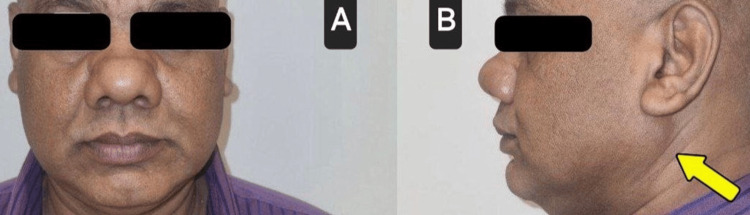
A preoperative photograph of the patient A: frontal view (no facial asymmetry is noted); B: profile view (presence of a mass inferior to left ear lobe is noted)

A 48-year-old male patient presented with a mass below the left ear lobe for the past two years. The patient had been experiencing a burning sensation on his left buccal mucosa for the past five months. The patient had a history of diabetes mellitus for the past six years, for which he was on medications. The patient was, upon examination, conscious, oriented, and afebrile. On extraoral examination, a 4 cm x 4 cm, non-tender, firm, non-pulsatile mass, roundish in shape, was palpated just inferior to the left ear lobe. The overlying skin was normal; that is, there was no sign of ulceration, erosion, or inflammation. No relevant findings were found upon intraoral examination. Upon inspection, there was no sign of any growth, swelling, or lesion. Upon palpation, the tissues were soft and smooth. There were no areas of tenderness palpated. From the clinical examination, a provisional diagnosis of pleomorphic adenoma of the left parotid gland was given (Figure 1).

**Figure 2:**
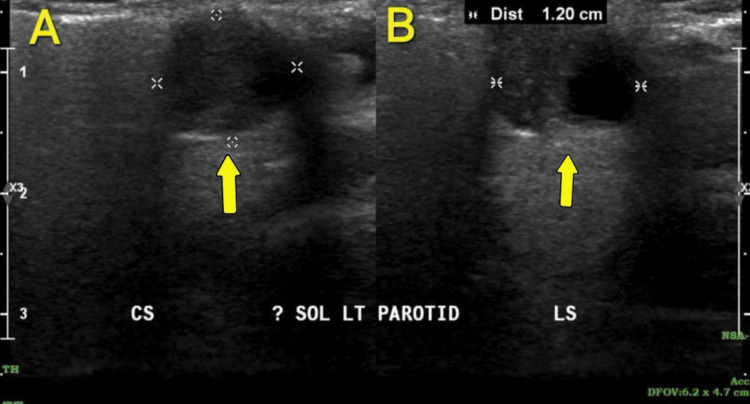
Ultrasound of the left parotid gland A: the coronal section (CS) shows a well-defined solitary hypoechoic lesion in the parotid gland; B: the longitudinal or transverse section (LS) also shows a hypoechoic lesion in the superficial lobe of the parotid gland.

Ultrasound (USG) was done for fine-needle aspiration cytology (FNAC) to be carried out directly from the mass in the left parotid gland of the patient (Figure 2).

**Figure 3:**
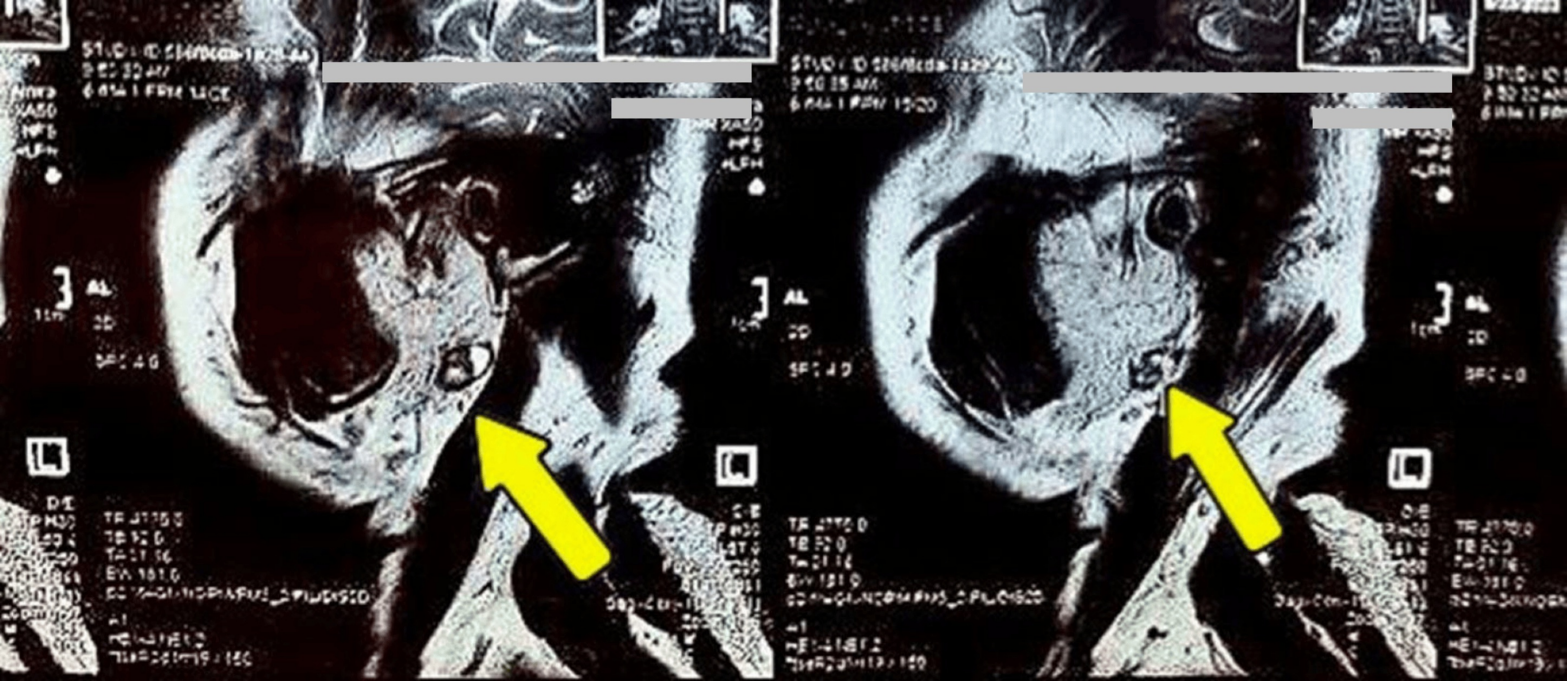
A preoperative MRI scan of the patient The figure shows two different sections of the sagittal view of the patient. The arrow shows the presence of a well-evident single ovoid mixed hyper-intense and hypo-intense area with well-defined margins in the superficial lobe of the left parotid gland.

A USG-guided FNAC was done. The report suggested a cellular smear, showing multiple variably sized cohesive clusters of cells with a scant to moderate amount of cytoplasm and uniform round nuclei. Occasional spindle cells and scattered fragments of the fibrillary chondromyxoid stroma without any atypical cells were noted. The features were suggestive of pleomorphic adenoma (The Millan System 2023, Category IV A benign low-grade neoplasm), which further confirmed the provisional diagnosis. An MRI was carried out to see the extent of the lesion (Figure 3).

**Figure 4:**
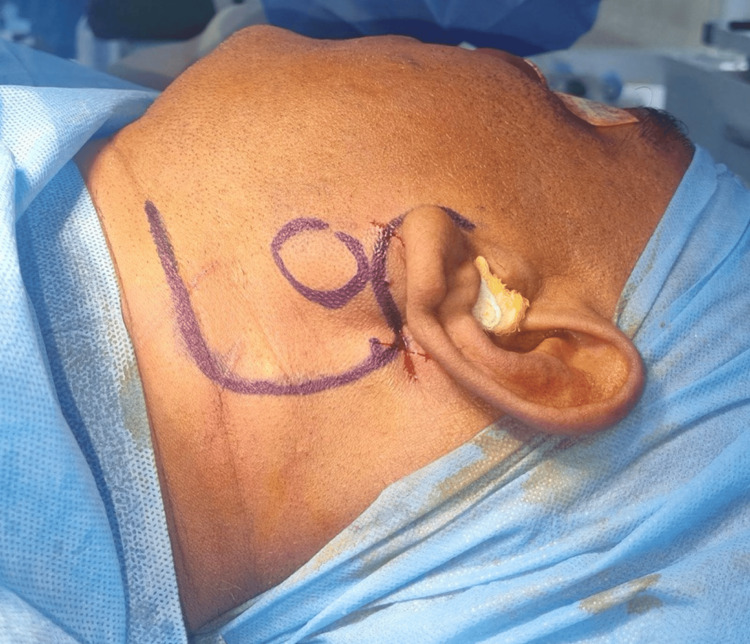
Incision markings

The patient was scheduled for a partial parotidectomy under general anesthesia. Under general anesthesia, naso-endotracheal intubation was done. Standard scrubbing and draping were done. A modified Blair’s incision was given on the left side. The incision extends anterior to the tragus of the ear as a preauricular incision. then it runs posteriorly to the mastoid tip, from where it runs inferiorly to join the neck crease (Figure 4).

**Figure 5:**
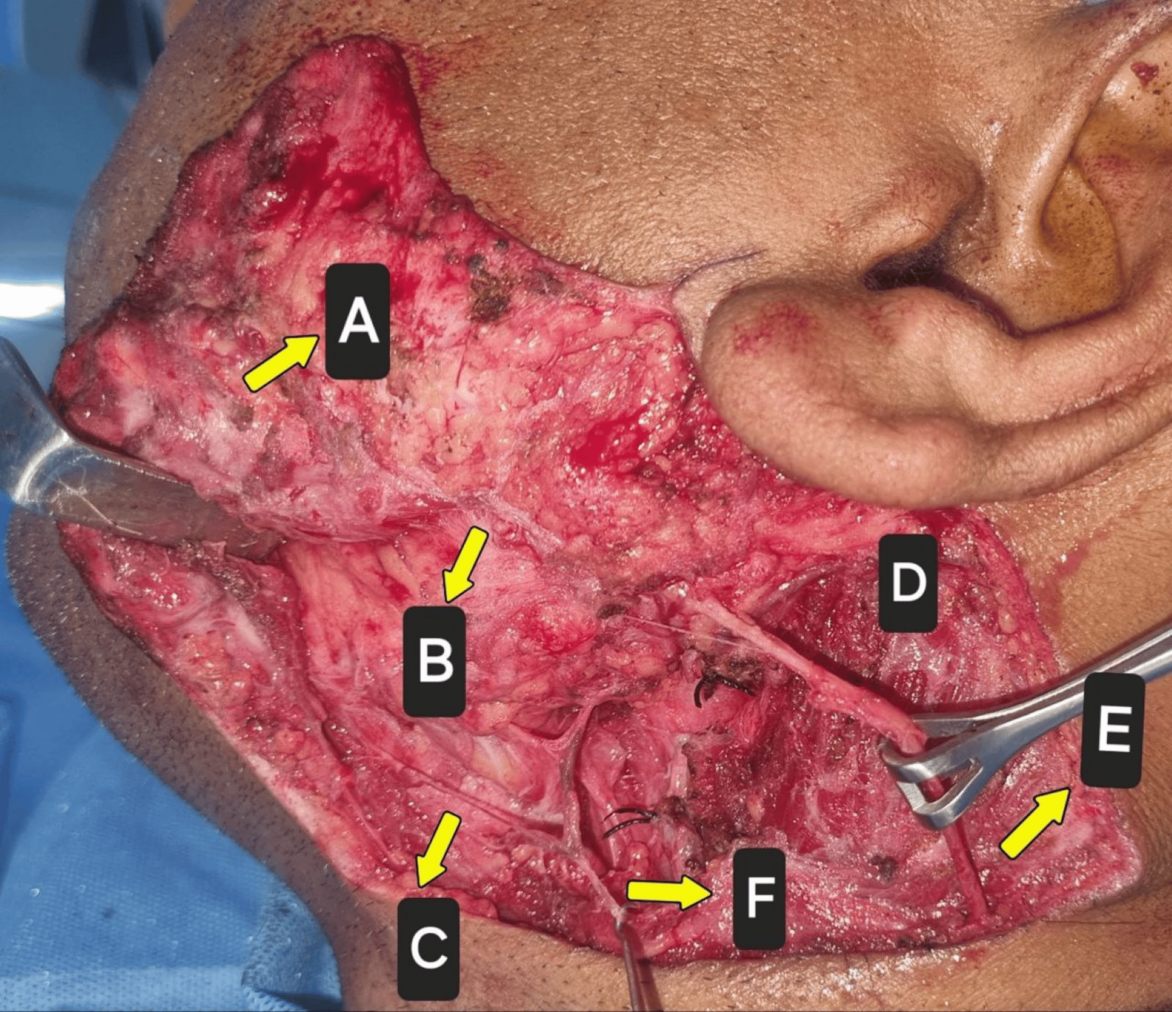
Sub-platysmal dissection was done and branches of the facial nerve were identified and preserved. A: platysma; B: buccal branch of the facial nerve; C: marginal mandibular branch; D: sternocleidomastoid muscle; E: greater auricular nerve; F: cervical branch of the facial nerve

A subplatysmal dissection was done. The branches of the facial nerve (buccal branch, marginal mandibular branch, and cervical branch) were identified and preserved. A partial parotidectomy was carried out, and the lesion was removed along with the superficial lobe of the parotid gland. Hemostasis was achieved. Closure was done in layers via 3-0 Vicryl and 3-0 ethilon (Figure 5).

**Figure 6:**
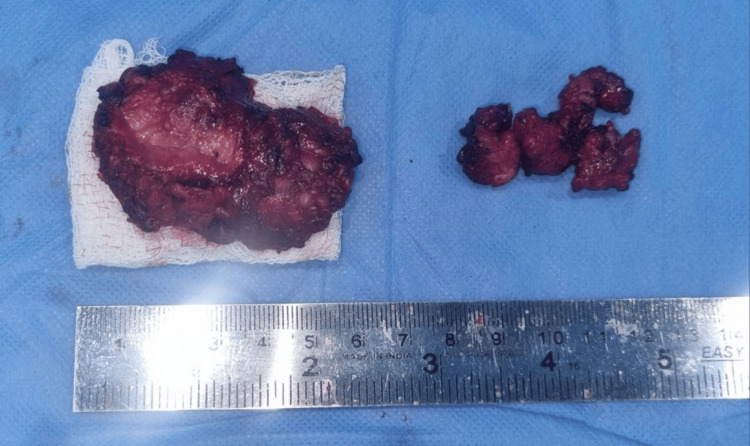
The excised specimen

After closure, extubation was done uneventfully and the specimen was sent for biopsy (Figure 6).

**Figure 7:**
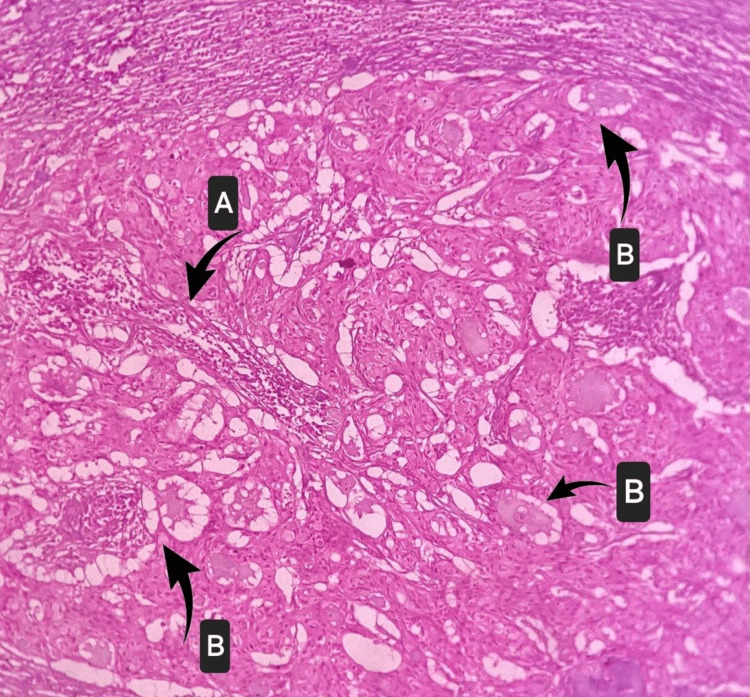
Histopathological examination (hematoxylin and eosin staining) under 20x A: connective tissue septum; B: lobules of cystic spaces

Upon histopathological examination, multiple hematoxylin and eosin (H&E) staining-stained sections showed partially encapsulated neoplasm of salivary gland origin composed of multiple lobules of variably sized cystic spaces separated by dense fibrous connective tissue stroma. The cystic spaces were lined predominantly by large oval to columnar-shaped cells with pale eosinophilic foamy cytoplasm and deeply basophilic nuclei, suggestive of mucous cells. In several areas, there was evidence of sheets of mucous cells admixed with polygonal-shaped cells with eosinophilic cytoplasm and hyperchromatic nuclei suggestive of epidermoid cells. A few clear cells were also seen. The cystic spaces were empty in a few areas, and in a few other areas, the cystic spaces were filled by abundant acellular basophilic to amphophilic material. The intervening stroma showed intense chronic inflammatory cell infiltration, moderate vascularity, and areas of hemorrhage. Multiple lobules of serous salivary gland acini, along with ductal structures, were also noted. There was evidence of overlying parakeratinized stratified squamous epithelium with pseudoepitheliomatous hyperplasia. Histopathology reveals malignant salivary gland neoplasms, MEC (low grade), and a sclerosing variant (Figure 7).

The patient has been on regular follow-ups for the past six months. No recurrence has been noticed since then.

Clinical differential diagnosis

In this case, there was left parotid swelling with a slight left ear lobe elevation. This is a typical sign of a benign parotid tumor, usually a pleomorphic adenoma. Another commonly occurring malignant salivary gland tumor with certain histological characteristics reminiscent of MEC is adenoid cystic carcinoma (ACC) [4]. An ACC usually shows a slower development rate and a proclivity for perineural invasion than MEC. Histologically, ACC is distinguished by a cribriform development pattern including basaloid cells and a distinct "Swiss cheese" look. Differentiating ACC from MEC can be accomplished with immunohistochemical markers like S100 and CK7 [5]. Another salivary gland tumor that can confuse us with MEC clinically is acinic cell carcinoma. Usually, it shows an acinar differentiation and a microcystic development pattern. Acinic cell carcinoma can be differentiated from MEC by immunohistochemical staining for antigens including lipase, amylase, and trypsin. A low-grade malignant salivary gland tumor, polymorphous low-grade adenocarcinoma (PLGA), might resemble MEC. A PLGA is confirmed histologically by a range of architectural forms, including cribriform, tubular, and solid development patterns. Differentiating PLGA from MEC can be accomplished using immunohistochemical staining for antigens including p63, S100, and smooth muscle actin [6]. Warthin tumor, also known as papillary cystadenoma lymphomatosum, is a non-cancerous tumor that develops in the salivary glands. It is characterized by a papillary cystic structure and the presence of oncocytic cells. Distinguishing between a Warthin tumor and MEC is crucial due to the distinct therapy and prognosis associated with each condition. Histopathological assessment and immunohistochemistry analysis can aid in differentiating between Warthin tumors and MEC [7].

## Discussion

Mucoepidermoid carcinoma is the most common salivary gland malignant tumor, comprising nearly 30% of all salivary gland malignancies [7]. Histologically, it consists of a combination of mucous-secreting, epidermoid (squamous), and intermediate cells [8]. Mucous cells are mucin-secreting and usually located within the tumor in cystic spaces. Epidermoid cells are squamous-like cells that may appear to form solid nests or sheets within the tumor. The intermediate cells possess characteristics of both mucous and epidermoid cells, which are seen in the tumor itself, but to a varying extent. Histologically, MECs are differentiated into low-grade (more mucinous), intermediate-grade, and high-grade on the basis of cell components in various proportions along with abnormal mitotic figures [9]. Low-grade tumors have a better prognosis, while tumors with a high grade are more aggressive, with a higher tendency for recurrences and metastasis. Patients with MEC may have a painless, palpable mass in the salivary glands, commonly in the parotid gland. Some patients may have other symptoms, like pain due to the compression of the facial nerve, facial asymmetry, or weakness of facial muscles.

Mucoepidermoid carcinoma is a broad family of malignant tumors of the salivary glands that demonstrate morphological heterogeneity and clinical variability [10]. Low-grade MEC is characterized by differentiated mucous, epidermal, and intermediate cells. Most low-grade MECs appear like well-demarcated cysts since such tumors are predominantly represented by mucin-producing cells [11]. Low-grade tumors have a better prognosis than high-grade neoplasms due to a slower growth rate and a low probability of reoccurrence and metastasis. Intermediate-grade MEC has a mix of well-differentiated and undifferentiated cells [12]. They exhibit features of both low and high grade, with a higher cellular atypia and mitotic rate than in low grade. Intermediate-grade has an inconsistent prognosis placed in between low-grade and high-grade tumors. High-grade MEC is poorly differentiated with undifferentiated cells, high mitotic rates, and cellular pleomorphism. It is characterized by aggressive behavior, including a higher tendency for local recurrence or distant metastasis and poor response when compared to low-level or intermediate-grade MEC. High-grade MEC commonly presents in progressive stages and requires more aggressive multimodal therapy. Clear-cell MEC is a rare subtype of MEC that contains clear cells with abundant cytoplasm [13]. Clear-cell MEC can be low, intermediate, or high-grade MEC and may have different tumor behaviors depending on the grading. The diagnosis of MEC is made based on clinical presentation as well as imaging (ultrasound, CT scan, or MRI) coupled with tumor tissue biopsy, followed by characteristic pathology features. Immunohistochemical studies are occasionally helpful to confirm the diagnosis and demonstrate antigenic similarities with other tumors, notably MEC [14].

Sclerosing MEC is the rarest variant that shows a sufficient amount of hyalinized or sclerotic stroma around tumor cells. The tumor cells are usually present as nests or cords among the fibrous stroma and can make a diagnosis especially difficult owing to their prominent features. Oncocytic MEC represents a third minor variation, in which oncocytoid cells have eosinophilic granular cytoplasm [15]. They can be composed of mucous, epidermoid, and oncocytic cells in various combinations, but histologically, they appear different from other variants. Sclerosing MEC is characterized by a nested or corded pattern with the presence of abundant fibrous stroma, and diagnosis may be difficult due to the predominant stromal component. It most commonly occurs in the parotid and minor salivary glands. It shows a prominent stromal sclerosis or fibrosis within the tumor [16]. Prominent stromal sclerosis, or fibrosis, is a salient feature of the sclerosing variant. The stroma is usually hyalinized and separates the epithelial elements. Tumor cells are generally positive for immunostains like cytokeratins, p63, or mucin stains. The diagnosis of sclerosing MEC consists of correlating the histological features, immunohistochemical staining, and clinical findings [17]. Quality diagnosis and identification of this subcategory are vitally important for superior patient treatment, owing to the small number of cases. The stroma may be dense, hyalinized, and contain collagen fibers, giving it a fibrotic or sclerotic appearance upon microscopy [18]. A similar case was reported in 2020, in a 23-year-old female in the United Kingdom. She was diagnosed with the same variant, for which she underwent superficial parotidectomy followed by radiation of 65 Gy in 30 fractions. As per the report published, the patient has been on a follow-up and has not had a recurrence as of now [19].

The treatment of MEC is essentially surgical resection. Surgery aims to excise the tumor with sufficient margins and retain as much non-involved tissue as possible, utilizing preservation for function due to salivary glands. Different factors can increase local recurrence (like high-grade tumors, positive margins after surgery, or lymph node involvement), and in such cases, adjuvant radiation therapy should be considered [20]. Mucoepidermoid carcinoma is not typically treated with adjuvant chemotherapy; however, it may be considered a stage IV or metastatic disease. The prognosis depends on the grade and stage of the diagnosis. Patients with low-grade tumors have a better prognosis and a higher survival rate, while high-grade tumors tend to recur and metastasize more commonly [21]. Patients with MEC should have regular follow-up care to monitor for recurrence or metastasis. Imaging studies may be done, blood tests may be taken, and the doctor will feel and look for any signs of the disease coming back. All patients with the sclerosing variant should receive a regular follow-up visit with the clinician. Follow-up is important because the side effects of treatment-that is, dry mouth, altered sense of taste, or damage to the facial nerve may occur [22].

## Conclusions

In conclusion, owing to the rarity of the sclerosing variant of MEC and the paucity of available data, the prognosis for this variant may be less well-known than for the more common variant. Factors determining the prognosis are likely to include these factors, as well as various other features. Therefore, close monitoring is important to optimize outcomes for patients with this variant. This case demonstrates the diagnosis and treatment of the rare sclerosing variant of MEC in a 48-year-old male. A correct diagnosis of the rare condition is possible, thanks to knowledge of its histologic features. Thus, it can be seen that the involvement of all the members of the multidisciplinary team (surgeons, pathologists, and oncologists) is of crucial importance for proper treatment and achieving the best possible outcomes. Finally, long-term follow-up is required because of the risk of the disease’s recurrence.

## References

[1] Devaraju R, Gantala R, Aitha H, Gotoor SG (2014). Mucoepidermoid carcinoma. BMJ Case Rep.

[2] Peraza A, Gómez R, Beltran J, Amarista FJ (2020). Mucoepidermoid carcinoma. An update and review of the literature. J Stomatol Oral Maxillofac Surg.

[3] Luna MA (2006). Salivary mucoepidermoid carcinoma: revisited. Adv Anat Pathol.

[4] Boahene DK, Olsen KD, Lewis JE, Pinheiro AD, Pankratz VS, Bagniewski SM (2004). Mucoepidermoid carcinoma of the parotid gland: the Mayo clinic experience. Arch Otolaryngol Head Neck Surg.

[5] Ide F, Obara K, Enatsu K, Mishima K, Saito I (2005). Sclerosing mucoepidermoid carcinoma of the oral cavity. J Oral Pathol Med.

[6] McHugh CH, Roberts DB, El-Naggar AK (2012). Prognostic factors in mucoepidermoid carcinoma of the salivary glands. Cancer.

[7] Sakamoto S, Kikuchi K (2024). Expanding the cytological and architectural spectrum of mucoepidermoid carcinoma: the key to solving diagnostic problems in morphological variants. Semin Diagn Pathol.

[8] Rapidis AD, Givalos N, Gakiopoulou H (2007). Mucoepidermoid carcinoma of the salivary glands. Review of the literature and clinicopathological analysis of 18 patients. Oral Oncol.

[9] Nance MA, Seethala RR, Wang Y, Chiosea SI, Myers EN, Johnson JT, Lai SY (2008). Treatment and survival outcomes based on histologic grading in patients with head and neck mucoepidermoid carcinoma. Cancer.

[10] Mimica X, Yuan A, Hay A (2021). Mucoepidermoid carcinoma: evaluating the prognostic impact of primary tumor site. Oral Oncol.

[11] Spellman J, Calzada G (2018). Mucoepidermoid carcinoma: a 23-year experience with emphasis on low-grade tumors with close/positive margins. Otolaryngol Head Neck Surg.

[12] Fadare O, Hileeto D, Gruddin YL, Mariappan MR (2004). Sclerosing mucoepidermoid carcinoma of the parotid gland. Arch Pathol Lab Med.

[13] Veras EF, Sturgis E, Luna MA (2007). Sclerosing mucoepidermoid carcinoma of the salivary glands. Ann Diagn Pathol.

[14] Tian W, Yakirevich E, Matoso A, Gnepp DR (2012). IgG4(+) plasma cells in sclerosing variant of mucoepidermoid carcinoma. Am J Surg Pathol.

[15] Heavner SB, Shah RB, Moyer JS (2006). Sclerosing mucoepidermoid carcinoma of the parotid gland. Eur Arch Otorhinolaryngol.

[16] Tasaki T, Matsuyama A, Tabata T, Suzuki H, Yamada S, Sasaguri Y, Hisaoka M (2013). Sclerosing mucoepidermoid carcinoma with eosinophilia of the salivary gland: case report and review of the literature. Pathol Int.

[17] Rasul U, Bradish T, Bashir MT, Shakeel M (2020). Sclerosing variant of mucoepidermoid carcinoma: a diagnostic challenge. BMJ Case Rep.

[18] Geisinger KR, Steffee CH, McGee RS, Woodruff RD, Buss DH (1998). The cytomorphologic features of sclerosing mucoepidermoid carcinoma of the thyroid gland with eosinophilia. Am J Clin Pathol.

[19] Shehadeh NJ, Vernick J, Lonardo F (2004). Sclerosing mucoepidermoid carcinoma with eosinophilia of the thyroid: a case report and review of the literature. Am J Otolaryngol.

[20] Harada H, Toyozumi Y, Sasaguri T, Kuyama K, Nakatsuka SI, Kurose A (2021). Sclerosing mucoepidermoid carcinoma of the salivary glands: report of three cases with special concern to the counterpart accompanied by eosinophilia. Med Mol Morphol.

[21] Sudhakar S, Velugubantla RG, Erva S, Chennoju SK (2014). Management of mucoepidermoid carcinoma of the palate utilizing (18)f-FDG PET/CT. J Clin Imaging Sci.

[22] Heptinstall L, Carroll C, Siddiqi J, Kamel D, Petkar M (2017). Sclerosing mucoepidermoid carcinoma of the submandibular gland presenting as chronic sialadenitis: a case report and review of literature. Head Neck Pathol.

